# Ylang-ylang and lavender aromatherapy for paediatric dental anxiety: A randomized control trial in first restorative appointment

**DOI:** 10.6026/973206300213686

**Published:** 2025-10-31

**Authors:** Amol N Patil, Sneha N Kamble, Pranali Shelke, Priya Telang, Aishwarya Shelar, Mrunalini Kadam

**Affiliations:** 1 Department of Paediatric and Preventive Dentistry, SMBT Denal College and Hospital, Sangamner, Maharashtra, India

**Keywords:** Dental anxiety, aromatherapy, lavender essential oil, ylang-ylang essential oil, paediatric dentistry, facial image scale, pulse rate

## Abstract

The safety and effectiveness of Ylang-ylang and Lavender essential oils in reducing anxiety in 6-10-year-old children during their first restorative dental appointment is of interest. Hence, sixty children were randomly assigned to one of three groups: Ylang-ylang, Lavender, or a placebo control, with anxiety measured by pulse rate and the Frankl Behaviour Rating Scale. Both aromatherapy groups showed a significant reduction in anxiety compared to the placebo, and Ylang-ylang specifically led to the greatest physiological calming effect as indicated by a larger decrease in pulse rate. Thus, we show that aromatherapy with either essential oil is a safe and effective non-pharmacological method for managing paediatric dental anxiety, supporting its use in clinical practice to improve children's initial dental experience.

## Background:

Dental fear and anxiety are significant challenges in pediatric dentistry, often leading to uncooperative behavior, delayed or avoided dental treatment and ultimately, poorer oral health outcomes [1]. The dental environment itself can be a source of anxiety, with stimuli such as the sight of needles, the sound of drilling, the smell of dental materials, and the sensation of high-frequency vibrations contributing to patient distress [2]. Studies have highlighted the powerful connection between odors and emotions, suggesting that olfactory stimuli can modulate mood and behaviour due to anatomical connections between brain structures involved in emotion and memory [2, 3]. In recent years, there has been a growing interest in complementary and alternative medicine (CAM) approaches, such as aromatherapy, for managing anxiety in medical and dental settings [4]. Aromatherapy involves the therapeutic use of essential oils-scented, volatile liquid substances derived from plants-to promote relaxation, well-being and healing through the sense of smell [5]. This method supports the concept that specific essential oils can elicit positive pharmacological and physiological effects. Lavender (Lavandula angustifolia) essential oil is widely recognized for its anxiolytic, sedative and mood-stabilizing properties. Its primary active constituents, linalool and linalyl acetate, are believed to interact with the gamma-aminobutyric acid (GABA) neurotransmitter system, contributing to its calming effects [6, 7]. Numerous studies have demonstrated lavender's efficacy in reducing anxiety across various clinical contexts, including surgical procedures, intensive care, and dental treatments [8, 9]. Ylang-Ylang (Cananga odorata) essential oil, extracted from the flowers of the Cananga odorata tree, is another essential oil traditionally used for its calming and uplifting qualities. Its chemical composition includes compounds such as germacrene D, farnesene and benzyl acetate [10]. Research indicates that ylang-ylang can reduce physiological markers of stress, such as blood pressure and heart rate and induce feelings of calmness, making it a potential agent for anxiety management [11, 12]. Despite the individual evidence supporting the anxiolytic properties of both lavender and ylang-ylang, a direct comparative evaluation of their effectiveness in reducing dental anxiety in a pediatric population, specifically during their first restorative treatment, is limited. Given the importance of anxiety control in pediatric dental care and the non-invasive, inexpensive nature of aromatherapy, this randomized controlled trial was designed to address this gap. Therefore, it is of interest to evaluate and compare the effect of aromatherapy using Ylang-Ylang flower essential oil and Lavender flower essential oil in reducing dental anxiety in 6-10-year-old children before and during their first restorative treatment appointment.

## Methodology:

This was a prospective, randomized controlled trial (RCT) conducted at the Postgraduate Clinical Section, Department of Paediatric and Preventive Dentistry, SMBT Dental College, Ghulewadi, Sangamner. The study was approved by the Institutional Ethics Committee. Written informed consent was obtained from the parents/guardians of all participants, and written informed assent was obtained from the participating children.

## Participants:

The study population comprised 6-10-year-old children visiting the Department of Paediatric and Preventive Dentistry for their first restorative treatment appointment. The calculated sample size was 60 participants, with 20 participants allocated to each of the three groups.

## Inclusion criteria:

[1] Normal healthy, well-nourished children aged between 6 and 10 years.

[2] Patients with prior parental consent and child assent.

[3] Patients who required restorative procedures.

[4] Patients undergoing dental treatment for the first time.

## Exclusion criteria:

[1] Children with common cold.

[2] Prior history of allergic reactions to aromatics.

[3] Impaired psychological development.

[4] Systemic diseases.

[5] History of an untoward experience in the medical setup due to any prevailing medical conditions.

[6] Any physical or mentally handicapping conditions.

## Randomization and blinding:

Participants fulfilling the inclusion criteria were selected and randomly allocated into three groups using the lottery method. This method ensured random assignment of participants to the control, lavender, or ylang-ylang groups. The study employed a double-blind design, where the participants were unaware of the specific intervention they received. The dental practitioners and outcome assessors were also blinded to the group allocation to minimize potential bias.

## Interventions:

All participants, irrespective of their group, were counseled for the restorative procedure using the "Tell-Show-Do" technique, a standard behavioral management approach in pediatric dentistry [13]. The three study groups were:

[1] Group I (Control): 20 Children subjected only to the "Tell-Show-Do" technique. No aromatherapy intervention was provided.

[2] Group II (Lavender Aromatherapy): 20 Children received aromatherapy using Lavender flower essential oil.

[3] Group III (Ylang-Ylang Aromatherapy): 20 Children received aromatherapy using Ylang-Ylang flower essential oil.

[4] For the aromatherapy groups (Group II and III), the essential oils were prepared as a 1% dilution (1 drop per teaspoon of carrier oil; 5-6 drops per ounce) using jojoba oil as the carrier. A fresh batch of diluted essential oil was diffused in the room using an aroma diffuser. The diffuser was activated for 15 minutes before the patient entered the dental setup and was then turned off. The patient was subsequently introduced to the dental environment and received "Tell-Show-Do" counselling for approximately 5 minutes, after which the restorative treatment commenced and was completed in approximately 15 minutes. Dental anxiety levels were assessed using two measures:

[5] Facial Image Scale (FIS)

[6] Pulse Rate

[7] Both FIS scores and pulse rates were recorded at:

[8] Pre-procedure: Before the child entered the dental setup.

[9] During procedure: While the aromatherapy was being used (for Groups II and III) during the first restorative appointment treatment.

## Results:

A total of 60 children, 20 in each group, completed the study. At baseline (pre-procedure), there were no statistically significant differences among the three groups concerning mean pulse rates (F=1.572, p=0.217) or Facial Image Scale (FIS) scores (F=0.158, p=0.854). This indicates that the groups were comparable at the commencement of the study.

An intra-group comparison of pulse rates as shown in Table 2 (see PDF) revealed varying outcomes. In Group I (Control), the mean pulse rate changed slightly from 77.6 ± 4.33 bpm before the procedure to 79.7 ± 6.75 bpm during the procedure, a non-significant reduction of -2.1 ± 6.78 bpm (t = -1.278, p = 0.138). In contrast, both treatment groups showed a significant decrease. Group II (Lavender) saw a mean pulse rate reduction from 79.6 ± 3.92 bpm to 76.2 ± 5.96 bpm, a significant decrease of 3.4 ± 8.13 bpm (t = 6.8, p = 0.031). Similarly, Group III (Ylang-Ylang) experienced a significant drop from 78.05 ± 2.79 bpm to 74.2 ± 4.5 bpm, a reduction of 3.85 ± 5.14 bpm (t = 4.9, p = 0.048). An inter-group comparison a s shown in Table 1 (see PDF) further highlighted the effectiveness of the aromatherapy treatments. There was a statistically significant overall difference in the reduction of pulse levels among the three groups (F=4.748, p=0.012). Post-hoc analysis using Tukey's test confirmed that the reduction in pulse rate was significantly greater in both the Lavender group (p=0.035) and the Ylang-Ylang group (p=0.02) compared to the Control group. Notably, there was no statistically significant difference in pulse rate reduction between the Lavender and Ylang-Ylang groups themselves (p=0.976). An intra-group comparison of FIS scores as shown in Table 4 (see PDF) showed no significant change in the Control group. Group I's mean FIS score shifted from 3.15 ± 0.93 to 3.1 ± 0.78, a non-significant reduction of 0.05 ± 0.94 (t = 0.6, p = 0.982). Both treatment groups, however, demonstrated significant improvements. In Group II (Lavender), the mean score significantly decreased from 3.25 ± 0.85 to 2.7 ± 0.86, a reduction of 0.55 ± 0.94 (t = 3.8, p = 0.03). Group III (Ylang-Ylang) also saw a significant decrease from 3.1 ± 0.78 to 2.7 ± 0.65, with a reduction of 0.4 ± 0.75 (t = 3.18, p = 0.048). The inter-group comparison of the reduction in FIS scores shown in Table 3 (see PDF) did not yield a significant difference. Overall, there was no statistically significant difference in score reduction among the three groups (F=1.679, p=0.196). Pairwise comparisons further confirmed this, showing no significant differences in FIS score reduction between the Control group and the Lavender group (p=0.184), the Control group and the Ylang-Ylang group (p=0.429), or between the two aromatherapy groups (p=0.854).

## Discussion:

This RCT investigated the comparative efficacy of inhaled Ylang-Ylang and Lavender essential oils in reducing dental anxiety among 6-10-year-old children undergoing their first restorative dental treatment. Our findings indicate that both essential oils significantly reduced physiological (pulse rate) and self-reported (FIS scores) anxiety within their respective groups from pre-procedure to during-procedure. Furthermore, the reduction in pulse rate was significantly greater in both aromatherapy groups compared to the control group, suggesting a beneficial anxiolytic effect of the essential oils. The significant intra-group reduction in pulse rate observed in both the Lavender and Ylang-Ylang groups aligns with the known physiological effects of these essential oils. Lavender, rich in linalool and linalyl acetate, is thought to exert its calming effects by interacting with the central nervous system, potentially modulating GABAergic activity [6, 7]. Similarly, Ylang-Ylang has been shown to reduce sympathetic nervous system activity, leading to decreases in heart rate and blood pressure [11, 12]. The significant inter-group differences in pulse rate reduction, where both aromatherapy groups outperformed the control group, further support the objective anxiolytic effect of these interventions in a dental setting. This is consistent with previous research demonstrating the efficacy of aromatherapy in reducing physiological signs of anxiety during dental procedures [4, 9]. A recent randomized controlled trial by El-Sayed *et al.* (2025) similarly reported that lavender-neroli oil aromatherapy significantly reduced heart rate and blood pressure in children during inferior alveolar nerve block anaesthesia [14]. Another study by Jafarzadeh *et al.* claimed that the use of aromatherapy with natural essential oil of orange could reduce salivary cortisol and pulse rate due to child anxiety state [15]. Regarding the self-reported anxiety measured by the Facial Image Scale (FIS), both Lavender and Ylang-Ylang groups showed a significant intra-group reduction in scores. This indicates that children in these groups perceived themselves as less anxious during the procedure after receiving the aromatherapy intervention. Interestingly, while the intra-group reductions were significant, the inter-group comparisons for FIS score reduction did not reach statistical significance. This could be attributed to several factors, including the subjective nature of the FIS, the relatively small sample size per group, or the possibility that the "Tell-Show-Do" technique in the control group also provided some level of anxiety reduction, thus narrowing the inter-group differences for self-reported measures. Previous studies have shown that behavioural management techniques like "Tell-Show-Do" are effective in managing paediatric dental anxiety [13]. The comparative efficacy between Lavender and Ylang-Ylang essential oils was not significantly different for either pulse rate or FIS scores. This suggests that both oils, when administered via inhalation at a 1% dilution, offer comparable anxiolytic benefits in this paediatric dental context. This finding provides flexibility for dental practitioners to choose between these two essential oils based on factors such as availability, cost, or individual patient preference for aroma. The study builds upon existing literature that supports the use of aromatherapy in dentistry [4, 5]. Specifically, Soni *et al.* [2] found that orange essential oil reduced anxiety and physiological parameters in children during their first dental visit, while Ghaderi and Solhjou [16] demonstrated lavender aromatherapy's ability to decrease dental anxiety and pain perception in children. Nirmala and Kamatham [17] also reported that lavender and sweet orange aromatherapy reduced dental anxiety in children undergoing local anesthetic administration. Tripathy *et al.* (2023) also found that both lavender and patchouli essential oils were effective in reducing anxiety scores and pulse rates in children, with lavender showing slightly better results [18]. Furthermore, research indicates that essential oils like lemon and ylang-ylang are effective in reducing anxiety symptoms, with ylang-ylang's anxiolytic effects potentially linked to the activation of 5-HTergic and DAergic pathways [19]. Another study by Abdalhai *et al.* (2025) demonstrated that inhaling lavender-neroli aromatherapy significantly reduced anxiety, heart rate, blood pressure, and pain in children during dental anesthesia procedures compared to controls. This evidence suggests lavender-neroli oil is a simple, safe, and effective method to ease pediatric dental anxiety and discomfort [20]. Moreover, study by Yadav *et al.* (2024) showed that essential oil aromatherapy, delivered before local anesthesia via nebulizer, led to significantly lower dental anxiety and pain scores in children compared to standard non-pharmacological techniques. Both anxiety and pain, measured on validated scales, were markedly reduced following the aromatherapy intervention, supporting its use as a safe and effective adjunct in pediatric dental care [21]. A comprehensive randomized controlled trial by Abdel Rehim *et al.* (2025) interrogated the anxiolytic potential of inhaled essential oils-lavender, chamomile, and peppermint-among children subjected to pulp therapy. The findings revealed that chamomile aromatherapy precipitated the most pronounced reduction in both pulse rate and anxiety scores, with lavender producing comparably substantial effects and peppermint yielding a moderate decrease in anxiety [22]. Our findings contribute to this body of evidence by directly comparing two distinct essential oils and providing both physiological and self-reported outcome measures in a randomized controlled trial setting with a paediatric population. Future research could explore optimal dosages and delivery methods (e.g., direct inhalation from a cotton ball during the procedure) for these essential oils in paediatric dentistry. Investigating the long-term impact of aromatherapy on children's dental anxiety and their acceptance of future dental treatments would also be valuable. Comparative studies with other non-pharmacological interventions or in combination with pharmacological methods could provide a broader understanding of their utility. Further research into the specific mechanisms of action of these essential oils in a clinical anxiety context would also be beneficial.

## Conclusion:

We show that aromatherapy, using either Lavender or Ylang-Ylang essential oil, is an effective and non-invasive method for significantly reducing dental anxiety in 6-10-year-old children during their first restorative dental treatment. Both essential oils showed comparable anxiolytic effects on both physiological (pulse rate) and self-reported (Facial Image Scale) measures. Data shows the integration of aromatherapy as a complementary strategy to enhance patient comfort and improve the dental experience for paediatric patients.
